# One-stop computerized virtual planning system for the surgical management of posterior wall acetabular fractures

**DOI:** 10.1186/s13018-022-03333-9

**Published:** 2022-10-04

**Authors:** Jianan Chen, Yifan Zheng, Zhixun Fang, Wei Zhou, Dan Xu, Guodong Wang, Xianhua Cai, Ximing Liu

**Affiliations:** 1 Department of Orthopedics, General Hospital of Central Theater Command, 627 Wuluo Road, Wuchang District, Wuhan City, Hubei Province China; 2 The First School of Clinical Medicine, Southern Medical University, Guangzhou City, Guangdong Province China

**Keywords:** Posterior wall, Acetabular fracture, Computerized, Preoperative planning

## Abstract

**Background:**

Posterior wall acetabular fractures remain one of the most difficult fracture injuries to treat. Accurate assessment of fracture characteristics and appropriate preoperative surgical strategies are essential for excellent reduction. This paper evaluates the feasibility and effectiveness of a one-stop computerized virtual planning system for the surgical management of posterior wall acetabular fractures.

**Methods:**

52 cases of posterior wall acetabular fractures treated surgically were selected in our department between January 2015 and December 2020 for retrospective analysis. 52 cases were classified into group A (25 patients) and group B (27 patients) according to whether computerized virtual planning procedures were performed preoperatively. In group A, virtual surgical simulation was conducted using a one-stop computerized planning system preoperatively. In group B, traditional surgery was employed. Reduction quality, surgical time, blood loss, hip function, complications, and instrumentation time were compared between the two groups.

**Results:**

The actual surgery for all patients in group A was essentially the same as the virtual surgery before the operation. Compared to group B, patients in group A had markedly shorter surgical time (−43 min), shorter instrumentation time (−20 min), and less intraoperative blood loss (−130 ml). However, no significant statistical difference was observed in reduction quality and hip function. The complication rate was slightly lower in group A (4/25) than in group B (7/27), without a significant difference.

**Conclusion:**

The one-stop computerized virtual planning system is a highly effective, user-friendly and educational tool for allowing the cost-efficient surgical simulation of posterior wall acetabular fractures and providing a more individualized therapeutic schedule. The one-stop computerized planning system is feasible to treat posterior wall acetabular fractures, which is an effective method than the conventional treatment of posterior wall acetabular fractures.

*Trial registration*: retrospective registration.

## Background

Posterior wall fractures are the most common type of acetabular fractures, approximately 1/4–1/3 of all acetabular fractures, which have worse results than most other patterns even in the hands of experienced surgeons [1–6]. Letournel and Judet [4] showed that acetabular fractures should be fully understood before treatment. It is regarded as an intra-articular fracture of a weight-bearing joint, and an accurate preoperative assessment of fracture characteristics and appropriate intraoperative reduction strategies as well as rigid internal fixation are necessary to ensure anatomical reduction in the articular surface for a satisfactory long-term hip function [4, 7, 8]. Therefore, a detailed preoperative surgical plan is essential for success.

Currently, three-dimensional computed tomography (3D-CT) has been widely used to identify fracture morphology and spatial relationship among bone blocks [9, 10]. While 3D-CT may improve the understanding of fracture morphology, it fails to offer complete insights into the actual configuration of fractures [11, 12]. In the traditional operation of posterior wall acetabular fractures, the fixation plates can be contoured after fractured segments are temporarily fixed with pins or screws. However, due to the morphological differences among individuals, achieving anatomical contouring intra-operatively will be time-consuming and difficult [13, 14]. The incomplete understanding of fracture patterns, as well as an imprecise plate preparation, may lead to a sub-optimal reduction [15].

Modern image processing and computer technology make it technically and financially easier for orthopedic surgeons to use virtual planning software in clinical practice [7, 16, 17]. In recent years, the application of computer-assisted virtual planning in complicated surgery of acetabular fractures has been reported with good clinical outcomes [13, 14, 16, 18]. However, the time consumed for creating virtual three-dimensional (3D) models and the software expertise needed to use this technology are the biggest obstacles to its widespread adoption [16, 19]. Hence, it is necessary to develop an efficient method for acetabular fracture fixation, requiring minimal effort with automatic and time-saving characteristics.

Herein, a one-stop computerized virtual planning system is proposed to enable a series of consecutive preoperative procedures including automatic segmentation, virtual fracture reduction, and internal fixation simulation via a personal computer by surgeons. This study evaluates the feasibility and effectiveness of the one-stop planning system for treating posterior wall acetabular fractures. Moreover, a retrospective comparison is conducted on the results of patients who suffer from posterior wall acetabular fractures with and without a virtual surgical planning system.

## Materials and methods

The Department of Orthopedics in the General Hospital of Central Theater Command performed a retrospective case-control study between January 2015 and December 2020. The Institutional Research Ethics Committee approved the study, and all participants provided written informed consent.

The inclusion criteria include: (i) isolated posterior acetabular wall fractures; (ii) age equal to or greater than 18 years. The exclusion criteria include (i) inability to undergo surgery within 3 weeks of injury; (ii) pathologic or open posterior wall acetabular fractures; (iii) history of hip injuries; (iv) along with pelvic or femoral head fractures; (v) patients with incomplete radiographic data and a follow-up time of fewer than 12 months.

52 cases with posterior wall acetabular fractures between January 2015 and December 2020 were selected for analysis. Patients were classified into groups A and B based on whether to perform the surgery by the new method of a one-stop computerized planning system or the traditional method, respectively. The new method consisted of 3D virtual fracture model reconstruction, reduction and implantation simulation. There were 25 patients in group A and 27 patients treated by the conventional technique in group B. Patient characteristics are summarized in Table 1, showing the comparability of demographics between both groups. All patients underwent obturator oblique, iliac oblique (Judet views) and anteroposterior (AP) pelvis views and CT scans (axial, 2D and 3D reconstruction) for detailed preoperative assessment. The closed reduction in the hip joint was performed at the emergency department under general anesthesia within 12 h of injury. For hip instability demonstrated on EUA (examination of the hip under anesthesia), tibial or femoral skeletal traction was performed after closed reduction to avoid re-dislocation and wear of the femoral head [2].

**Table 1:** Baseline characteristics of patients

Variables	Group A (*n* = 25)	Group B (*n* = 27)	Test value	*P* value
Age (years)	46.64 ± 11.07	42.81 ± 11.72	t = 1.208	0.233
*Gender*				
Male	16	17	*χ*2 = 0.006	0.938
Female	9	10		
*Mechanism of injury*				
Fall from height	6	5	*χ*2 = 0.272	0.873
Traffic accident	16	19		
Other injuries	3	3		
*Fracture side*				
Left	15	16	*χ*2 = 0.003	0.957
Right	10	11		
*Concomitant injuries*				
Yes	9	9	*χ*2 = 0.041	0.840
No	16	18		
Hip dislocation	17	18	*χ*2 = 0.010	0.918
Preoperative sciatic nerve damage	5	4	*χ*2 = 0.244	0.621
Time to surgery (days)	9.12 ± 3.59	8.26 ± 2.68	*t* = 0.985	0.329

### Computerized virtual planning procedure

In group A, thin-slice CT axial images of the pelvis were output to the DICOM file and input to the computerized virtual system (3D Image Workstation; Wei Zhuo Zhi Yuan Ltd, Beijing, China) on a personal computer.

**Fig. 1:**
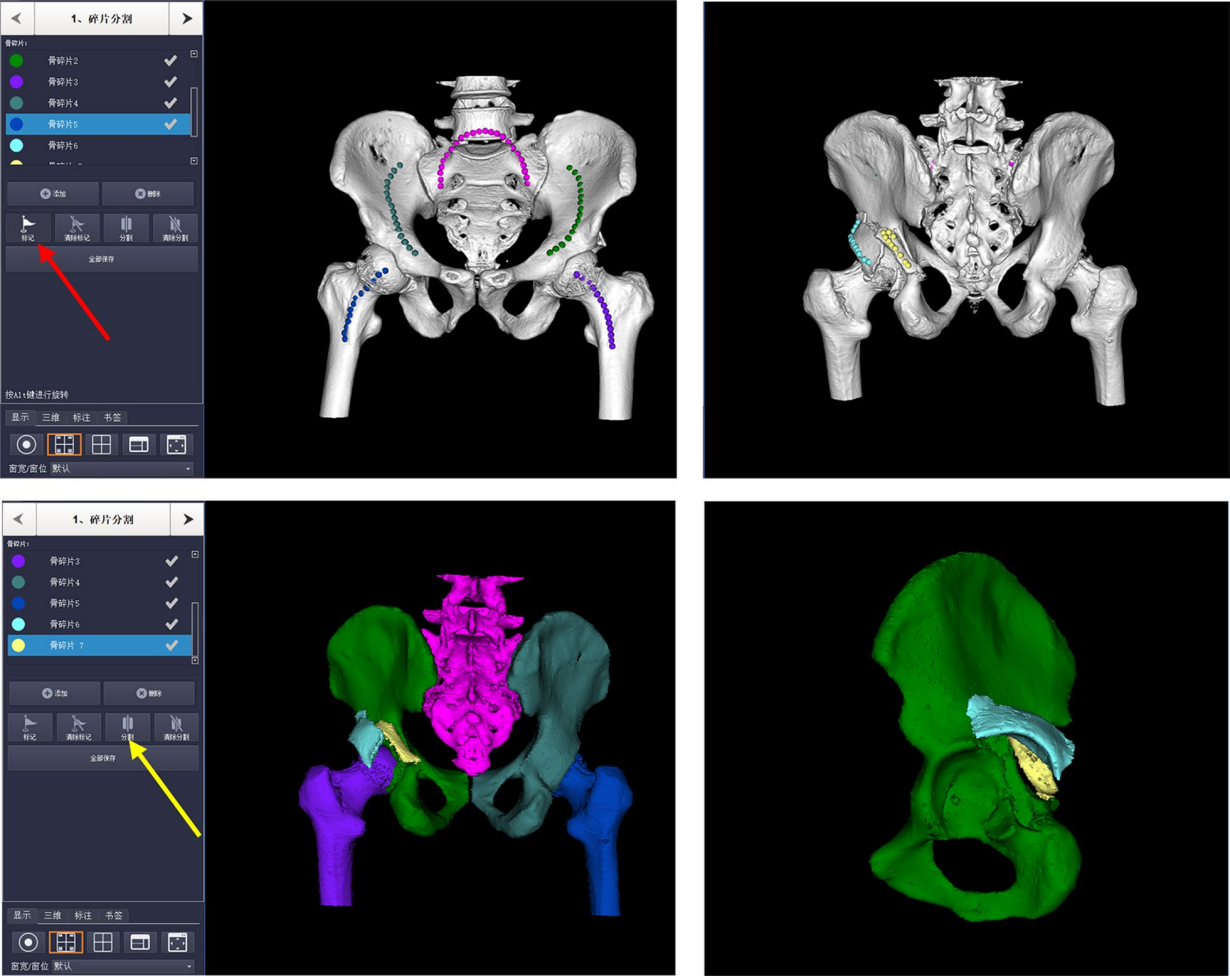
3D automatic segmentation for the virtual fracture model. The approximate extent of each part was marked with different colors on the 3D fracture model using the marker function (red arrow). After clicking the “segmentation key” (yellow arrow), the software segmentation processing started and then adjacent fragments were separated as individual parts in different colors (light blue, green, and yellow represented three individual fragments in the left acetabulum)

**Fig. 2:**
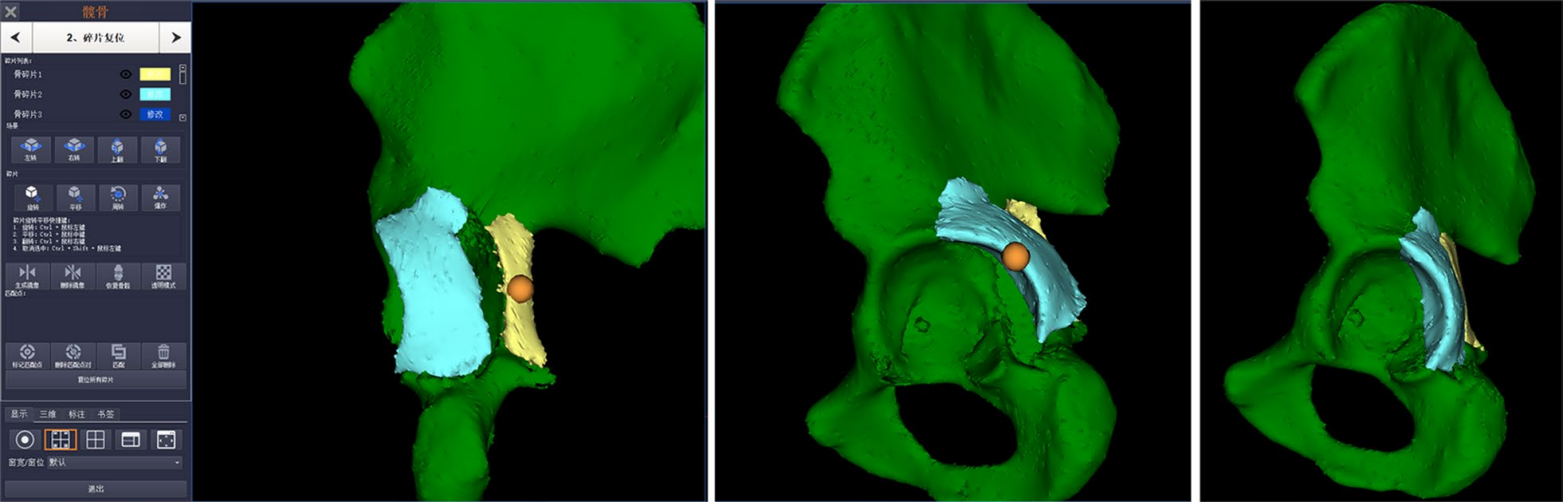
Simulation of fracture reduction. An anatomical reduction in all three planes was obtained by moving and rotating bone fragments

**Fig. 3:**
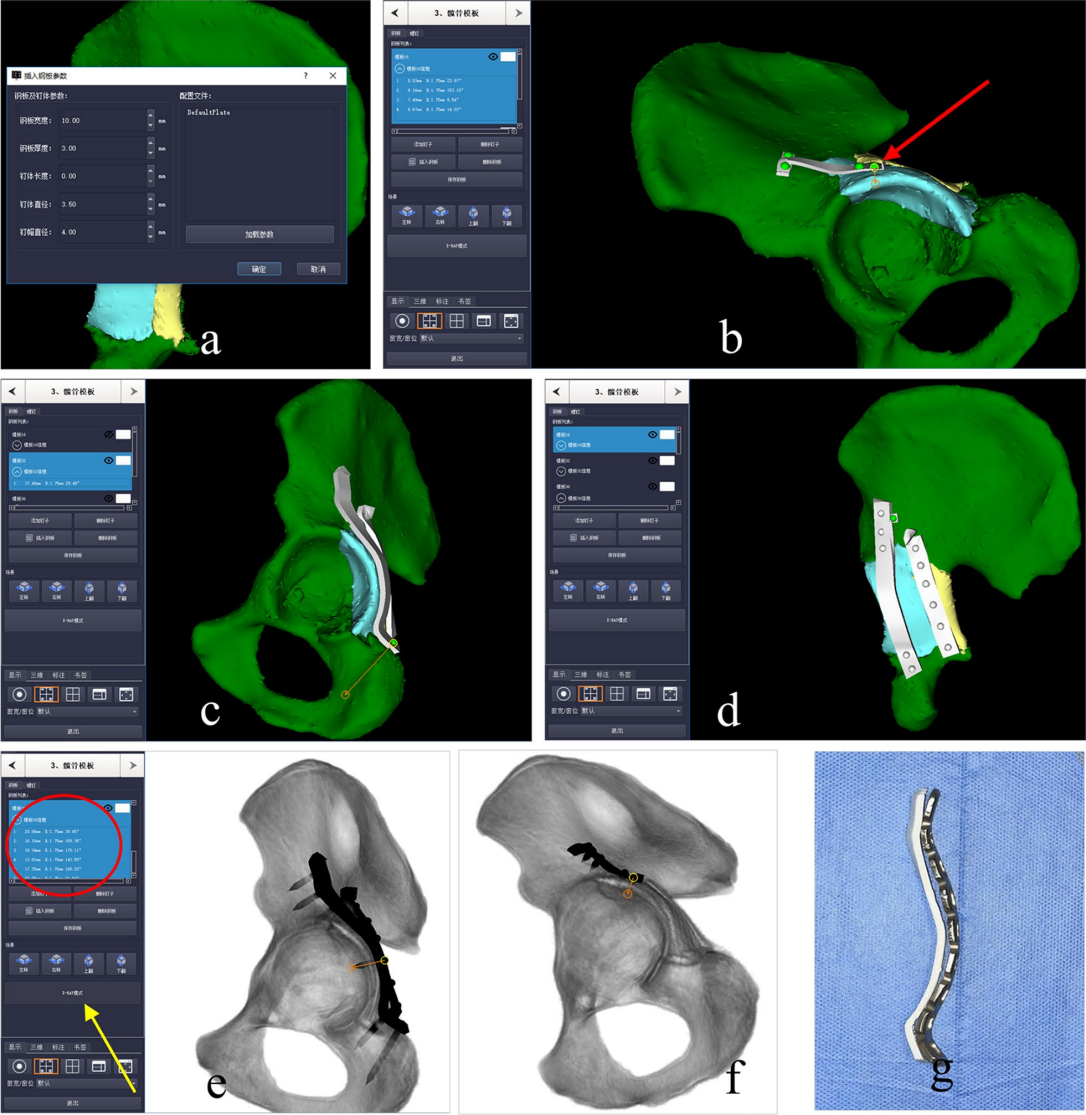
Simulation of internal fixation. **a** Fixation parameters were preset, including the width and thickness of plates and screw diameter. **b** The miniplate was put on the acetabular roof and the screw length near the acetabular rim was measured (red arrow). **c**–**d**. The reconstruction plates were automatically contoured onto the virtual bone surface and the required screws were inserted. **e**–**f** After the “X-ray model” (yellow arrow) was clicked, the screw placement was further confirmed to avoid intra-articular penetration and the precise length of the screw was recorded (red circle). **g** The reconstruction plates were contoured based on the 3D-printed template and then sterilized for surgical preparation

The one-stop preoperative planning process on software was as follows: Firstly, the appropriate threshold for bone was measured by default and a virtual 3D model of the pelvis was reconstructed. Then fracture fragments were separated using a 3D automatic-segmentation function. The approximate extent of each part was approximately marked using the device cursor, followed by a software algorithm precisely and automatically defining the fragment border when adjacent fragments were defined as separated parts. Different colors were then assigned to each fracture fragment to make it easier for surgeons to identify different fragments (Fig. 1). Following reconstruction and segmentation, a post-reduction model was obtained by moving and rotating each separated bone fragment in all three planes, through which the key anatomical landmarks and the best sequential procedures for fracture reduction can be determined. Furthermore, detailed spatial relationships can be observed by removing bone fragments and femoral head (Fig. 2). After virtual anatomical reduction, the “fixation key” was clicked to outline the shape of miniplates and reconstruction plates on the 3D post-reduction model, after which the tetrahedral model of desired plates was automatically contoured onto the virtual bone surface according to these sketches with preset fixation parameters. Next, the required screws were chosen and inserted precisely into the plates by rotating the virtual anatomically reduced model and changing its transparency to avoid intra-articular screw penetration under the X-ray model. In this study, miniplates like phalangeal and metacarpal plates were routinely adopted for posterior wall acetabular fractures. Lastly, the desired reconstruction plate templates designed on the virtual post-reduction model were exported as stereolithography (STL) files and 3D printed using poly-lactic acid (PLA). The 3D-printed plate templates were used as a reference to contour patient-specific reconstruction plates before surgery, during which the destruction of the screw hole should be avoided carefully (Fig. 3). The time required to complete these steps was recorded including software time, 3D-printing time, and plate preparation time.

### Surgical procedure

All patients were placed laterally on a radiolucent table and all operations were conducted under general anesthesia by two experienced trauma surgeons. In both groups, the Kocher–Langenbeck (K–L) approach was used to expose the posterior wall.

**Fig. 4:**
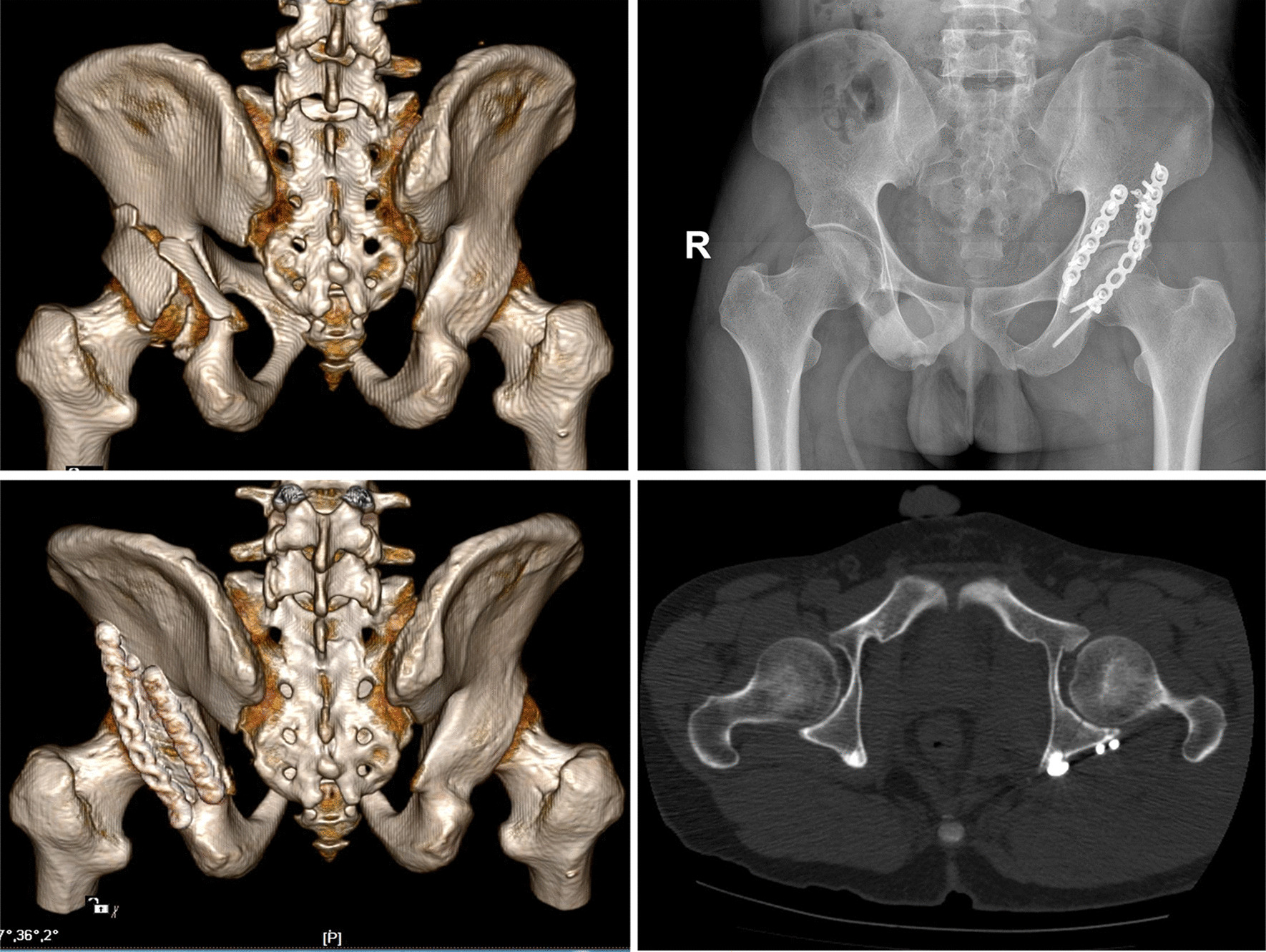
The outcomes of virtual preoperative planning were identical to the actual internal fixation mode and the cross-sectional CT image showed an anatomical reduction according to the Matta grading score

**Fig. 5:**
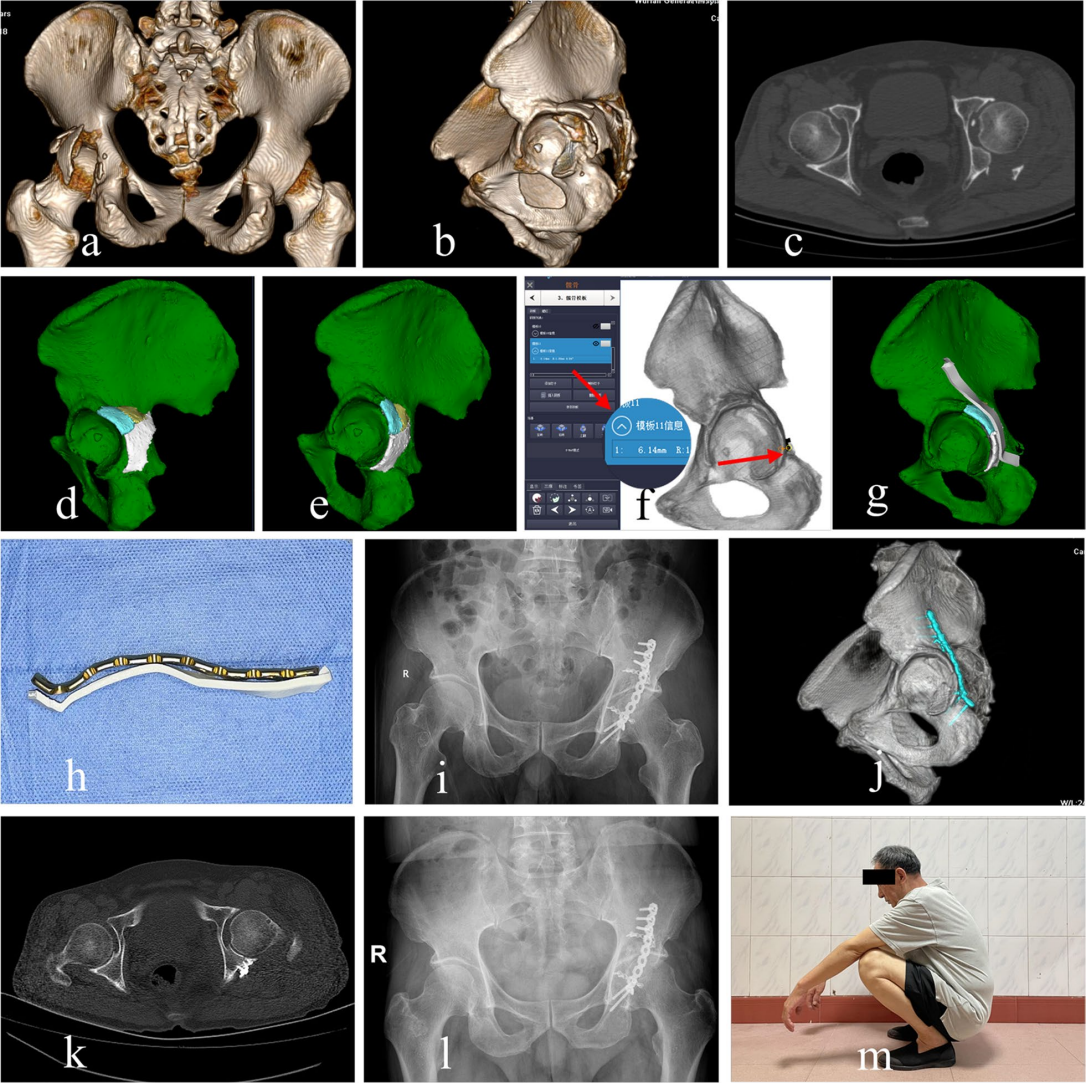
A 62-year-old man with posterior wall acetabular fractures underwent preoperative surgical planning using the one-stop computerized virtual planning system. The preoperative 3D-CT (**a**, **b**) and cross-sectional CT image (**c**) showed a comminuted fracture of the posterior wall of the left acetabulum with significant displacement. The left acetabulum with separate fragments (**d**) was 3D reconstructed virtually and then reduced anatomically (**e**). Virtual simulation of internal fixation (**f**, **g**) was conducted on the computer and the length of the mini-screw in dangerous areas was also measured (red arrow). Then, patient-specific reconstruction plates were ready for intra-operative placement with the aid of 3D-printed templates (**h**). Postoperative AP view (**i**), 3D-CT (**j**) and cross-sectional CT image (**k**) showed an anatomical reduction, with the mini-screw in a good position. Postoperative AP view (**l**) and the range of motion (**m**) at 30 months after surgery

In group A, aided by detailed preoperative planning, key anatomical landmarks were precisely exposed with less invasiveness and appropriate sequential procedures for fracture reduction were performed. After adequate fracture reduction, miniplates were placed in the pre-determined position, and then fixation was enhanced by the patient-specific reconstruction plates to improve its stability and prevent postoperative fixation loss (Fig. 4). The number and position of miniplates depended on the comminution of posterior wall fragments and the distribution of fracture lines. A typical case is shown in Fig. 5.

In group B, the fracture nature was learned by surgeons to make the surgical plan using plain radiographs and 3D-CT scans conventionally. The same surgical procedure was used in this group of patients. The miniplates and reconstruction plates were placed empirically without the assistance of one-stop planning system. The reconstruction plates were all contoured intraoperatively, and the time required for implantation was recorded. Fracture reduction and positioning of miniplates and reconstruction plates were confirmed using intraoperative fluoroscopy in both groups.

### Post-operative management

The same management was conducted in both groups. Prophylactic antibiotic (cefazolin sodium) was routinely administered and continued for 48 h postoperatively. The drainage amount was recorded continuously and the drainage tube was removed at the 48th hour postoperatively. All the patients were treated with low molecular weight heparin (LMWH) as anticoagulation therapy. Routine chemoprophylaxis for heterotopic ossification was not used. 3D-CT of the pelvis and standard pelvic plain films (Judet and AP views) were obtained within one-week post-operation and rehabilitation training was guided by rehabilitation doctors for all patients after surgery. All patients were followed up at 1, 2, 3, 6, and 12 months after surgery and annually thereafter. During the follow-up, postoperative complications, clinical function, and fracture healing were recorded.

### Result evaluation

Fracture reduction quality, complications, blood loss, instrumentation time, and surgical time were assessed. Instrumentation time refers to the time needed for plate fixation, including fixing plate configuration adjustments, drilling screw holes, measuring screw lengths, and locking plates. Based on the criteria of Matta, the quality of posterior wall fracture reduction was expressed as poor (> 3 mm), imperfect (2–3 mm), and anatomic (0–1 mm) [20]. During the follow-up, the Modified Merle d’Aubigné score was adopted to evaluate clinical outcomes [21]. The score was graded as poor (< 13), fair (13 or 14), good (15, 16, or 17), and excellent (18).

### Statistics

SPSS software version 20.0 and student t-test were used for continuous variables. Continuous variables with a normal distribution were expressed as the mean ± standard deviation. Categorical variables were expressed as relative (%) and absolute (*n*) frequencies. Chi-square and Wilcoxon rank-sum tests were used for Categorical variables as appropriate. *P* value < 0.05 was defined as statistical significance.

## Results

### Clinical data

Demographic data, duration of surgery, preoperative sciatic nerve damage, hip dislocation, concomitant injury, and fracture side are presented in Table 1. No significant difference was found in preoperative variables between the two groups (*p* > 0.05). The mean follow-up periods were 28.24 ± 8.13 and 29.56 ± 7.63 months in groups A and B (*p* = 0.550). No difference was found in the fracture healing time between the two groups (15.48 ± 2.31 vs. 16.37 ± 2.39 weeks for groups A vs. B, *p* = 0.179).

### Peri-operative clinical parameters

The preoperative software time was 24.92 ± 3.70 min in group A. 3D printing time for the virtually designed plate templates was 125.80 ± 11.06 min. The time for patient-specific plate preparation was 10.44 ± 1.69 min. The average blood loss and surgical time in group A were markedly lower than those in group B (426.40 ± 141.93 vs. 555.93 ± 138.28 ml, *p* = 0.002; 138.64 ± 42.37 vs. 182.26 ± 47.99 min, *p* = 0.001). It was also found that the mean instrumentation time of group A was markedly shorter compared to group B (27.92 ± 10.84 vs. 48.19 ± 15.96 min, *p* < 0.001). According to the Matta scoring system, in group A, anatomic, imperfect, and poor grades were observed in 22 (88.0%), two (8.0%), and one (4.0%) cases, respectively. In group B, anatomic, imperfect, and poor grades were observed in 21 (77.8%), four (14.8%) and two (7.4%) cases, respectively. The quality of fracture reduction was similar between both groups (*P* = 0.337) (Table 2).

**Table 2:** Surgical and clinical outcomes

Variables	Group A (*n* = 25)	Group B (*n* = 27)	Test value	*P* value
Software time (min)	24.92 ± 3.70			
3D printing time (min)	125.80 ± 11.06			
Plate preparation time (min)	10.44 ± 1.69			
Blood loss (ml)	426.40 ± 141.93	555.93 ± 138.28	*t* = −3.332	0.002
Surgical time (min)	138.64 ± 42.37	182.26 ± 47.99	*t* = −3.463	0.001
Instrumentation time (min)	27.92 ± 10.84	48.19 ± 15.96	*t* = −5.314	0.000
*Quality of reduction*				
Anatomic	22 (88.0%)	21 (77.8%)	*z* = −0.960	0.337
Imperfect	2 (8.0%)	4 (14.8%)		
Poor	1 (4.0%)	2 (7.4%)		
*Hip function*				
Excellent	16 (64.0%)	15 (55.6%)	*z* = −0.620	0.535
Good	7 (28.0%)	9 (33.3%)		
Fair	1 (4.0%)	2 (7.4%)		
Poor	1 (4.0%)	1 (3.7%)		
*Complications*				
Yes	4 (16.0%)	7 (25.9%)	*χ*2 = 0.767	0.381
No	21 (84.0%)	20 (74.1%)		
Heterotopic ossification	2 (8.0%)	3 (11.1%)		
Posttraumatic arthritis	2 (8.0%)	3 (11.1%)		
Avascular necrosis of femoral head	0	1 (3.7%)		

### Post-operative hip function

Based on the modified Merle d’Aubigné score, in group A, the function results at the final follow-up were graded as poor in one case (4.0%), fair in one (4.0%), good in seven (28.0%), and excellent in 16 (64.0%). In group B, the function results at the final follow-up were graded as poor in one case (3.7%), fair in two (7.4%), good in nine (33.3%), and excellent in 15 (55.6%). No significant statistical difference was found in the hip function outcomes between both groups (*P* = 0.535) (Table 2).

### Post-operative complications

Posttraumatic arthritis occurred in five patients, two (8.0%) of group A and three (11.1%) of group B. In group A, no patient developed avascular necrosis of the femoral head after the operation. In group B, one (3.7%) patient developed avascular necrosis of the femoral head and was treated with total hip arthroplasty one year postoperatively. Based on the Brooker classification, five patients developed the class I heterotopic ossification (HO) with no clinical symptoms (two in group A and three in group B). No perioperative complications including incision infection, iatrogenic sciatic nerve injury, and deep vein thrombosis occurred in either group. None of the patients had plate failure, screw loosening, miniplate displacement, or intra-articular screw placement at the follow-up.

## Discussion

The acetabular fracture is caused by severe intra-articular trauma, frequently from high-velocity injury [2, 3]. The primary purpose of displaced acetabular fractures in the weight-bearing area is the anatomic reconstruction of the articular surface to recover the congruity of the hip joint and rigid internal fixation to allow for rapid postoperative recovery with early rehabilitation [1, 4–6]. However, the treatment of posterior wall acetabular fractures remains a relatively challenging task for orthopedic surgeons because of deep osseous geometry, limited direct visualization with close-set neurovascular structure and various fracture patterns like comminution or marginal impaction areas [5, 22]. Previous studies have suggested that despite the use of straightforward reduction and internal fixation techniques, a significant proportion of patients undergoing surgical treatment obtain poor clinical outcomes. In Letournel’s series of 117 isolated posterior wall fracture subgroups, 18% of patients undergoing open reduction and internal fixation had poor results [23]. Matta [20] also reported in his retrospective study that 32% of patients with posterior wall fractures had poor clinical outcomes after surgery. Similarly, Saterbak et al. [22] reported that 35% of patients with posterior wall fractures had complete loss of joint space in one year postoperatively. Articular reduction is highly correlated with functional results in treating posterior wall acetabular fractures [2, 4, 5, 20, 21]. In recent years, the internal fixation of posterior wall acetabular fractures has achieved significant progress, but these fixations remain in the initial stage and cannot be widely implemented in clinical practice [24–26]. Accurate assessment of fracture characteristics, adequate preoperative surgical planning, and custom-made plates and screws can ensure the success of acetabular surgery, especially for younger and less experienced surgeons [13–16, 27]. Therefore, meticulous preoperative surgical planning for the treatment of posterior wall acetabular fractures may be beneficial in obtaining precise anatomical reduction to lower the risk of post-traumatic arthritis and achieve good clinical outcomes.

During the past decade, virtual preoperative planning of orthopedic surgery based on a virtual reality model has become increasingly common with the development of computer technology and image processing [7, 17, 19, 27]. Based on the real CT data, Hu et al. [8] utilized computerized virtual simulation for acetabular fracture reduction and plate fixation, which could help surgeons to better understand fracture patterns and determine correct surgical strategies. Later, some scholars suggested that the combination of computerized virtual planning procedures and the 3D printing-assisted contoured plate fixation method is a valuable tool for surgeons to formulate an appropriate preoperative plan in acetabular fracture surgery [11–13, 15]. Although blood loss and surgical time were significantly reduced under the aid of preoperative planning, they had the same common limitations such as technical expertise required for software operation and time consumed in preparing a full size (1:1) 3D-printed hemipelvis model, which would prevent the widespread use of this technique. Therefore, a one-stop computerized virtual planning system was utilized in this study to overcome the above-mentioned demerits. There are several noteworthy characteristics of the one-stop planning system in this study. In previous studies [13, 15, 18], the segmentation of bone fragments required manual operation on all 2D CT slices in all three planes, which was time-consuming and needed software expertise. In this study, a more efficient segmentation method based on a 3D virtual model was used to separate contiguous bone fragments, which necessitated no special knowledge of computer technology and could be operated by orthopedic surgeons themselves. Furthermore, digital plate models must be complicatedly designed and imported from multiple software programs before the virtual simulation of internal fixation [7, 16, 17]. Through such a one-stop computerized virtual surgical planning system, a sequence of preoperative plans, including segmentation, fracture reduction and virtual internal fixation, could be completed quickly within 30 min. The average time for using the software was 18 min in group A, which was much shorter than that reported by Hu et al. (79 min). Additionally, the plate template designed on the virtual post-reduction model was 3D printed using our methodology, instead of a full-sized 3D-printed hemipelvis model. The time needed for a full-sized (1:1) 3D-printed hemipelvis model was 12 h as reported in recent studies, but only 2 h for a plate template in our series, which significantly reduced the time and cost.

This study is unique in case and control groups where patients with similar fracture types were included, who were surgically treated by the same Kocher–Langenbeck approach. Thus, the interference factors could be reduced as much as possible with a higher level of reliability. This study aims to compare the outcomes of the posterior wall acetabular fractures treated by the one-stop preoperative planning system and the traditional method. Compared to the conventional surgical method in group B, both surgical time and blood loss in group A were significantly reduced by the computerized virtual planning system. The decreased surgical time was mainly due to preoperative surgical simulation in a 3D virtual model. Psychological studies have suggested that cognitive preparation and mental readiness are necessary for successful procedures, especially for beginners [7]. Given that the preoperative surgical planning system allows for the preoperative knowledge of fracture morphology, surgeons can avoid extensive soft-tissue dissection and perform fracture reduction more easily, which significantly reduced intraoperative blood loss. Consequently, psychological and physical requirements on surgeons were reduced, as well as risks associated with prolonged general anesthesia. Previous studies have demonstrated that patient-specific plate preparation in acetabular fracture surgery can avoid intraarticular screwing and improve the quality of fracture reduction, especially for comminuted fractures [11–15]. Aided by the 3D-printed plate template designed on the computerized system in group A, the plate can be accurately contoured to achieve optimal compactness with the bone surface, ensuring mechanical strength and reduction accuracy as well as shortening instrumentation time significantly. Moreover, the length of mini-screws in the 3D virtual model was measured to enhance the safety of screw placement, particularly at fixation points in dangerous areas. It is noteworthy that differences between real and virtual conditions should be kept in mind for surgeons. Bone fragments would not be attached to soft tissues in a virtual environment, so plates and screws are easily placed in any direction. However, soft tissues would interfere with reduction and narrow the working space, especially when faced with severely comminuted fractures. In group A, there was one case in group A presented with severely comminuted fractures obtained a poor reduction quality, though an appropriate surgical plan was formulated preoperatively. The planned strategies for reduction had minor changes as soft tissues prevented the fragment manipulation in the desired fashion. Nevertheless, in this study, higher hip-function scores and fracture reduction quality were found in posterior wall acetabular fractures treated by the computer-assisted virtual planning system, although these differences were not significant. In summary, with the aid of the one-stop computerized virtual planning system, the same fracture reduction quality and functional results were obtained with less surgical time and blood loss. Besides, there are educational benefits for young surgeons to enhance the learning curve for understanding fracture patterns and surgical treatment of posterior wall acetabular fractures.

There are also some limitations in the present study. First, the process of repositioning fragments is manual, which is still not precise and fast enough, especially for fractures with severe comminution. Further study may better address this problem by using the mirrored image from the opposite acetabulum, which may be more efficient. Second, the shape of the virtual contoured plate is too ideal to bend the real plate because the destruction of the screw hole should be avoided when bending. Therefore, the virtual contoured plate may not match each brand of the reconstruction plates. Third, the present study is a retrospective design with a relatively small sample size. Further prospective randomized controlled trials with a large sample size may ascertain the role of this one-stop computerized virtual planning system in the surgical treatment of posterior wall acetabular fractures.

## Conclusion

The one-stop computerized virtual planning system is an efficient, user-friendly and educational tool, which allows cost-efficient surgical simulation of posterior wall acetabular fractures and provides a more individualized treatment plan. With the assistance of a one-stop planning system, shorter surgical and instrumentation time, and less blood loss were observed during the surgery of posterior wall acetabular fractures. Consequently, the one-stop planning system is feasible to treat posterior wall acetabular fractures, which is an effective method than the conventional treatment of posterior wall acetabular fractures.

## Data Availability

The datasets used or analyzed during the current study are available from the corresponding author on reasonable request.

## Abbreviations

2D: Two-dimensional
3D: Three-dimensional
HO: Heterotopic ossification
DICOM: Digital Imaging and Communications in Medicine
PLA: Poly-lactic acid
AP: Anteroposterior
STL: Stereolithography
EUA: Examination of the hip under anesthesia
